# Predictors of Segmented School Day Physical Activity and Sedentary Time in Children from a Northwest England Low-Income Community

**DOI:** 10.3390/ijerph14050534

**Published:** 2017-05-16

**Authors:** Sarah L. Taylor, Whitney B. Curry, Zoe R. Knowles, Robert J. Noonan, Bronagh McGrane, Stuart J. Fairclough

**Affiliations:** 1 Physical Activity and Health Research Group, Department of Sport and Physical Activity, Edge Hill University, St. Helens Road, Ormskirk, Lancs L39 4QP, UK; Whitney.Curry@edgehill.ac.uk (W.B.C.); Robert.Noonan@edgehill.ac.uk (R.J.N.); Stuart.Fairclough@edgehill.ac.uk (S.J.F.); 2 Physical Activity Exchange, Research Institute for Sport and Exercise Sciences, Liverpool John Moores University, Liverpool L3 2AT, UK; Z.R.Knowles@ljmu.ac.uk; 3 School of Arts Education & Movement, Dublin City University Institute of Education, St. Patrick’s Campus, Dublin, Ireland; bronagh.mcgrane@dcu.ie; 4 Department of Physical Education and Sports Science, University of Limerick, Limerick, Ireland

**Keywords:** physical activity, schools, children, accelerometer

## Abstract

Background: Schools have been identified as important settings for health promotion through physical activity participation, particularly as children are insufficiently active for health. The aim of this study was to investigate the child and school-level influences on children′s physical activity levels and sedentary time during school hours in a sample of children from a low-income community; Methods: One hundred and eighty-six children (110 boys) aged 9–10 years wore accelerometers for 7 days, with 169 meeting the inclusion criteria of 16 h∙day^−1^ for a minimum of three week days. Multilevel prediction models were constructed to identify significant predictors of sedentary time, light, and moderate to vigorous physical activity during school hour segments. Child-level predictors (sex, weight status, maturity offset, cardiorespiratory fitness, physical activity self-efficacy, physical activity enjoyment) and school-level predictors (number on roll, playground area, provision score) were entered into the models; Results: Maturity offset, fitness, weight status, waist circumference-to-height ratio, sedentary time, moderate to vigorous physical activity, number of children on roll and playground area significantly predicted physical activity and sedentary time; Conclusions: Research should move towards considering context-specific physical activity and its correlates to better inform intervention strategies.

## 1. Introduction

Physical activity (PA) is associated with numerous health benefits in school-aged children [1]. Beneficial effects relate to cardiovascular [2] and cardiometabolic risk factors [3], and mental health [4]. Internationally it is recommended that children engage in moderate-to-vigorous PA (MVPA) every day for at least 60 min [5,6,7]. Report cards on the overall PA of children and youth across 38 countries using self-reported data from a number of surveys have specified that levels are low [8]. Grades of D- were given to England, Australia, Canada and USA, indicating that less than 30% of children in these countries are sufficiently active [8]. Moreover, data from the International Children′s Accelerometry Database (ICAD) [9] reveal that children aged 4–18 years engage in MVPA for an average of 30 min per day [10], and that after the age of 5 years there is an average decrease of 4.2% in total PA with each additional year of age, due to lower levels of light-intensity PA (LPA) and also a progressive increase in the volume of sedentary time (ST) [9]. Excessive time spent sedentary is positively associated with markers of adiposity and cardiometabolic risk [11]. International PA guidelines make further recommendations in regards to limiting the amount of ST children accrue [5,6,12]. Current evidence suggests that screen time has a bigger impact on health compared with overall ST [13]. For example, television viewing has been shown to demonstrate a strong relationship with overweight/obesity and inverse relationships with fitness [14]. High levels of time engaging in screen-based ST have also been linked to lower self-esteem in youth [15].

This evidence linking low PA and high ST to adverse health outcomes warrants interventions which promote PA participation and ST reduction in children. Within the school setting there are PA opportunities during discretionary periods between lessons and at break times/recess, through classroom activities, during structured PA periods such as physical education (PE) lessons, and through extra-curricular opportunities before and after the formal school day [16]. Investigations have indicated that PA during school recess can contribute towards up to 40% of a child′s recommended daily PA [17], whilst PE has been shown to play a substantial role in providing PA for children as they are more active on days with PE than without [18]. Thus, schools have been identified as a key environment for child PA promotion. Over 95% of youth and therefore the full socio-economic spectrum of the paediatric population can be reached and engaged regardless of individual circumstances [19,20,21]. Lower socioeconomic status (SES) home environments typically provide more opportunities for ST and fewer for PA [22]. It has been argued that more positive attitudes towards the value of PA and healthy lifestyles are evident in families with a higher SES, which may be reflected by high SES children attaching greater importance to PA participation for health benefits, relative to perceptions from a comparable group of low SES children [23]. This trend was observed by Drenowatz et al. [24], through the use of household income as an indicator of SES, and steps per day to assess free-living PA, with lower PA levels and more time in sedentary behaviours found among low SES children. However, use of different methods of measuring PA and SES suggest that associations reported between SES and children′s PA are equivocal [25]. School environments provide the opportunity for SES influence to be minimised due to all children attending regardless of individual circumstances. In order to develop effective PA interventions within schools it is important to understand all factors which influence participation [26]. PA and sedentary behaviours are complex and their occurrence varies within different domains. Youth PA and ST correlates are represented at the individual, interpersonal, organisational, and system levels [27]. In addition to SES, correlates consistently associated with PA in children include sex, age, ethnicity, perceived competence, and perceived barriers [28]. Whilst it is useful to understand what influences children′s habitual PA and ST, these may not be consistent within specific contexts and environments such as schools [29] and thus their investigation is warranted.

Schools are identified as important settings for health promotion through PA. In the UK, the Government′s plan for action to reduce childhood obesity has reinforced the importance of school recommending that children should accumulate at least 30 min of MVPA within school every day [30]. For schools to be active environments and for successful interventions to be implemented, it is important to understand what influences PA-related behaviour during school hours. The aim of this study therefore, was to investigate the child and school-level influences on children′s PA levels and ST during school hours in a sample of English children from a low-income community.

## 2. Materials and Methods

### 2.1. Participants

Seven primary schools participated in the baseline phase of the cross-sectional Active Schools: Skelmersdale (AS:Sk) study. The schools were located in Skelmersdale which is situated within the West Lancashire borough of North-West England. The percentage of children living in income-deprived households within this area (34.6%) is above the national average for England (21.8%) and average overweight and obesity prevalence in 10–11 year olds exceeds 33% [31]. All 15 schools in the town were invited to participate in the project. Twelve schools initially expressed interest and were provided with more details, which resulted in seven schools consenting to take part. Reasons given by schools that declined to participate included lack of time to commit to the three phases of the project, and uncertainty as to whether they would be able to get parental consent for a sufficient number of children. Once ethical approval from the Faculty of Arts and Sciences Research Ethics Committee at Edge Hill University was granted (SPA-REC-2015-183), the schools received the relevant paperwork to invite all Year 5 children (ages 9–10 years; n = 243) to participate in the study. Returned signed parent/carer consent and child assent forms were received from a sample of 215 children aged 9–10 years (88% participation rate).

### 2.2. Child-Level Measures

*Anthropometry.* Stature was assessed to the nearest 0.1 cm using a portable stadiometer (Leicester Height Measure, Seca, Birmingham, UK). Body mass was assessed to the nearest 0.1 kg (761 scales, Seca). Body mass index (BMI) was calculated as body weight in kilograms divided by height in meters squared for each participant. BMI z-scores were assigned [32] and age and sex specific BMI cut points established children as normal weight or overweight/obese (those who were underweight were grouped into the normal weight category) [33]. Gender-specific equations were used to predict children′s age from peak height velocity (APHV), as a proxy measure of biological maturation [34]. Waist circumference was measured to the nearest 0.1 cm using an anthropometric tape measure, and the percentage of waist circumference-to-height ratio (%WHtR) was calculated as a measure of central adiposity [35]. All measurements were conducted on school sites by the lead author and a research assistant using standard procedures.

#### 2.2.1. Socio-Economic Status

Neighbourhood-level SES was calculated using the 2015 Indices of Multiple Deprivation (IMD) [36]. The IMD is a UK Government produced deprivation measure for England comprising income, employment, health, education, housing, environment, and crime. IMD rank scores were generated from parent-reported home post codes using the National Statistics Postcode Directory database. IMD rank scores were matched to their corresponding IMD deciles, where decile 1 represents the most deprived 10% of areas nationally.

#### 2.2.2. Psychological Outcomes

Children′s perceptions of PA self-efficacy and enjoyment were assessed through a paper questionnaire pack. Questions were completed by children in class time under the guidance of a class teacher, teaching assistant and at least two research assistants. Teachers were asked to indicate any children with reading or comprehension issues who were then provided with one-to-one support. Included was eight items measuring self-efficacy [37] and 16 items measuring enjoyment [38], which were measured on a 5-point scale ranging from 1 (“Strongly disagree”) to 5 (“Strongly agree”). These questionnaires have previously demonstrated strong factorial validity [37,38].

#### 2.2.3. Cardiorespiratory Fitness

The 20 m shuttle run test was conducted to provide an estimate of cardiorespiratory fitness (CRF) [39]. This well-established test has been previously used with children of a similar age to those in the current study [40,41]. The total number of shuttles completed by each participant was recorded as a proxy measure of CRF.

#### 2.2.4. Physical Activity

Children wore an ActiGraph GT9X triaxial accelerometer (ActiGraph, Pensacola, FL, USA) on their non-dominant wrist for seven consecutive days. Children were instructed to wear the accelerometer all the time (24 h⋅day^−1^) except when engaging in water-based activities such as bathing and swimming. The ActiGraph GT9X accelerometer uses the same validated MEMS sensor as the ActiGraph GT3X+ model [42] which has been used extensively in child PA research [43]. Log sheets were provided for children to record times when the accelerometer was removed and replaced. Data collection took place during the regular school term from May to July 2016 therefore data were representative of usual spring/summer free-living activities. Accelerometers were initialised to record raw accelerations at a frequency of 30 Hz. After 7 days of wear, accelerometer data were downloaded using ActiLife version 6.11.8 (ActiGraph) and saved in raw format as GT3X files. These were subsequently converted to CSV format to facilitate raw data processing. Files were processed in R (http://cran.r-project.org) using the package GGIR (version 1.1–4). GGIR converted the raw triaxial accelerometer signals into one omnidirectional measure of acceleration termed the Euclidean norm minus one (ENMO; vector magnitude taken from the three axes minus the value of gravity with negative values rounded up to zero) [44,45]. ENMO values were averaged per 1 s epoch over each of the seven monitored days [46].

Accelerometer non-wear was determined using the method of van Hees et al. [44], which has been applied previously in ActiGraph studies involving children [46,47,48]. Briefly, non-wear time was estimated from the standard deviation and value range of each accelerometer axis, calculated for moving windows of 60-min with 15-min increments [44]. Accelerometer wear time inclusion criteria were at least 16 h·day^−1^ for a minimum of three weekdays [49]. This minimum wear time criteria is sufficient to produce reliable estimates of PA [50]. After children without sufficient wear time were excluded from the data set, there was an analytical sample of 169 children, whose descriptive characteristics did not differ from those of the excluded children. Published ENMO prediction equations were used to identify cut-points for classifying activity into ST, LPA, and MVPA [51]. Previously, children′s ST has commonly been defined as being equivalent to 1.5 METs based on standard MET-based definitions in adults [52]. Better classification accuracy for differentiating ST (from LPA) has though been reported using 2 METs which accounts for the higher energy expenditure of children relative to adults [52]. Therefore, the Hildebrand equations were solved for 2 METs (ST/LPA) and 4 METs (MVPA) resulting in ENMO cut-points of 33 mg for LPA, and 370 mg for moderate PA (MPA), respectively. Sleep was estimated within the GGIR R package (version 1.2–11; http://cran.r-project.org). Briefly, nocturnal periods of time where there was no change in arm angle greater than 5 degrees over at least 5 min, were classified as sleep periods [53].

### 2.3. School-Level Measures

#### 2.3.1. School PA Provision Survey

Head teachers or the most appropriate alternate member of staff from each school completed a 20-item survey to indicate school PA environment, practices, and provision. The survey was available to complete online or in paper format. Three existing U.S.-based PA audit tools were used and adapted to create UK-culturally appropriate questions (i.e., School Physical Activity Policy Assessment; School Health Index) [54,55]. Questions covered various parts of the school day relating to PA, including the amount of provision before and after school as well as aspects relating to recess and PE lessons. A 4-point scale was used to answer questions (0–3), with a score of 3 representing optimal PA environment/practice/provision and 0 representing poor or non-existent PA environment/practice/provision. The item scores were summed, divided by 60, and converted to percentage scores.

#### 2.3.2. Playground Space

Aerial views of the schools′ playground areas were located using the Google™ Earth Pro application (version 7.1). Playground areas were calculated using the polygon tool and summed for each school to provide an estimate of playground spatial area [56,57]. The number of enrolled children in each school (number on roll) was obtained from school records.

### 2.4. Data Analysis

Individual and school level descriptive statistics (mean ± SD) were calculated for all measured variables. Independent t-tests assessed sex differences in the main outcomes of ST, LPA, and MVPA. To account for the clustering of children within the seven schools, multilevel modelling was performed for the main analysis using MLwiN Version 2.02 [58]. A 2-level data structure defining children as the first level unit of analysis and schools as the second level unit was used [59]. Separate multilevel prediction models with random intercepts were constructed to identify significant predictors of ST, LPA, and MVPA during the school day (range 8.45 am–3.15 pm), morning break (mean 15.7 min), lunch break (time on the playground only, mean 37.9 min) and total PE time (mean 90.7 min; 12 models in total). Morning break and lunch break periods were daily occurrences for all participating schools, PE frequency differed between schools and was either once or twice per school week. School- and child-level predictors were entered into the models and were retained when they were significantly associated with the outcomes and remained significant when subsequent predictors were added to the models. Therefore, non-significant predictors which were not in the final models were not presented in the results. Regression coefficients in the models were assessed for significance using the Wald statistic and the alpha level was set at *p* < 0.05 [59].

## 3. Results

### 3.1. Exploratory Analyses

The descriptive characteristics of the 215 children are displayed in Table 1. Around one-quarter of the children were classified as overweight or obese. The deprivation deciles of home postcodes ranged from 1 to 9, with 85% of children living within deciles 1–3. One hundred and eighty-six children met the wear time inclusion criteria (87% compliance) and were subsequently included in the main analyses. Table 2 presents the mean number of minutes spent in different PA intensities during weekdays, indicating that boys and girls did not achieve the recommended 60 min of MVPA on average. The mean number of minutes spent in the different PA intensities across the studied segments (school day/morning break/lunch break/PE) are also presented in Table 2.

### 3.2. Main Analyses

School-level predictors entered into the multilevel models were number of enrolled students, playground area, and PA provision score (Table 3). Only six out of seven schools were included for the PA provision scores due to non-completion of the survey by one school. The multilevel analyses are reported in Table 4, Table 5, Table 6 and Table 7.

### 3.3. School Day Predictors

The only correlate to significantly predict school day ST was school day MVPA levels (*p* < 0.001), whereby one minute of MVPA during the school day predicted 1.9 min less ST during the same period (*p* < 0.001). Participation in school day ST predicted less participation in LPA (0.9 min, *p* < 0.001) and MVPA (0.1 min, *p* < 0.001) during the school day. CRF (*p* < 0.001) and number on roll (*p* = 0.01) were also inverse predictors of school day LPA. Conversely, CRF was a positive predictor of school day MVPA (*p* < 0.001), while maturity offset was an inverse predictor of school day MVPA (*p* < 0.001). Out of school MVPA was a significant inverse predictor of LPA in the school day (*p* < 0.001) and a significant positive predictor of MVPA in the school day (*p* < 0.001).

### 3.4. Morning Break Predictors

MVPA during the school day predicted less ST participation during morning break (*p* < 0.001). ST during the school day also predicted less morning break LPA (*p* < 0.001) and MVPA (*p* < 0.001) but by only 0.1 min. Out of school MVPA predicted less participation in LPA during morning break (*p* = 0.02). Number on roll positively predicted ST (*p* = 0.01) and LPA (*p* < 0.001) at morning break. Those who were overweight or obese participated in significantly less MVPA during morning break (*p* = 0.01), and maturity offset was also an inverse predictor of MVPA (*p* < 0.001).

### 3.5. Lunch Break Predictors

MVPA during the school day predicted less ST participation during lunch break (*p* < 0.001). ST during the school day also predicted less lunch break LPA (*p* < 0.001) and MVPA (*p* < 0.001). Out of school MVPA predicted more MVPA participation during lunch break (*p* = 0.002). Number on roll was a positive predictor of both ST (*p* = 0.045) and MVPA (*p* < 0.001) during lunch break. WtHR predicted less MVPA during lunch break by 9 min (*p* < 0.001).

### 3.6. PE Lesson Predictors

Inverse relationships were evident between school day MVPA and ST during PE (*p* < 0.001), as well as school day ST and LPA (*p* < 0.001) and MVPA (*p* < 0.001) during PE. Overweight or obese children engaged in significantly more LPA during PE than normal weight children (2.6 min, *p* = 0.001). Further positive predictors of PE MVPA were PA enjoyment (*p* < 0.001) and out of school MVPA (*p* < 0.001), while maturity offset was an inverse predictor of MVPA during PE lessons (*p* < 0.001).

## 4. Discussion

This study investigated predictors of low-income children’s school environment PA levels and ST. Significant child-level predictors were maturity offset, CRF, weight status, WtHR, ST, and MVPA, while the significant school-level predictors were number of children on roll and playground area. Previous research has reported variables such as sex, SES, and self-efficacy to be predictors of children’s habitual PA [28]. However, these predictors were not associated with PA or ST during the whole school day or specific segments of the school day in this study. The fact that SES was not a significant predictor was likely due to the homogeneity in the children’s IMD scores. The exploration of children’s time-specific PA has identified age and gender to be consistently associated with school morning break PA [29]. Significant differences were observed between boys and girls for school day ST and MVPA, for MVPA during morning break and PE, and for lunch break ST, LPA, and MVPA in the current study, but sex was not significantly related to ST or PA in the multilevel analyses. Previous research has shown the effect of sex on PA to reduce or even disappear when maturity status is controlled for [60,61]. This research may explain why sex did not predict ST and PA, but maturity offset significantly predicted MVPA during the school day, morning break, and PE. Disengagement from PA aligning with maturation is associated with a variety of behavioural, social and biological factors [62]. Furthermore, the contribution of biological maturity to variation in PA should consider factors such as activity context [62]. Our results indicate that children’s maturity status influences MVPA in the school environment, thus it is important to understand how school PA practices and policies recognise this influence to enable all children to engage in MVPA during school hours regardless of their maturity status. Furthermore, the children in this study were largely pre- and early-pubescent. The influence of maturation may be exacerbated in high school environments as PA is known to gradually decline as adolescents progress toward the mature state, i.e., adulthood [63].

Sedentary time and MVPA were the most consistent predictors across the different periods, with MVPA significantly predicting less ST, and ST levels significantly predicting less MVPA. This is consistent with previous research studying break time periods of the school day, in which an inverse association was reported between sedentary activities and percentage of time engaged in MVPA [64]. Whilst our analysis found that one behaviour predicted less of another, this does not imply that ST displaces PA and vice versa. Marshall and colleagues [65] found correlations between sedentary behaviours and PA to be small and positive, suggesting ST does compete with and coexist with PA. However, small increases in MVPA levels within the school environment which help to reduce ST should be advocated due to the known health and development benefits of MVPA and negative health implications of excessive ST in children [13]. The replacement of sedentary behaviour with PA is also of particular importance for children who are overweight or obese. Weight status was a significant predictor in the current study, with those who were overweight or obese participating in less MVPA during morning break for example. Results from intervention studies suggest that preventing excessive sedentary behaviour may be an effective approach in improving healthy weight among children [66]. As overweight/obese children have a higher chance of becoming overweight or obese as adults and subsequently being at risk for chronic diseases [67], advocating reduced ST and increased MVPA in the school setting among this group is important. Additionally, out of school MVPA was a significant inverse predictor of LPA during the school day, morning break and PE, and a significant positive predictor of MVPA during the school day, lunch break and PE. Given that activity during the school day was low overall, it appears that children who accrued more MVPA out of school participated in more during school, regardless of individual schools′ PA provision. Conversely, creating more opportunities for activity during the school day can prompt higher activity levels to be sustained out of school, which lends further support for promoting MVPA participation in the school setting [68].

A significant predictor of MVPA during PE lessons was PA enjoyment. This reinforces the need for children′s PA experiences to be fun and enjoyable as PA enjoyment is a recognised mediator of behavioural change in PA interventions [69]. This finding aligns with theories of motivation, in that the participation in activities for joy or pleasure results in a greater adherence due to participants being intrinsically motivated to engage [70]. Enjoyment is a key principle of the recently proposed ”SAAFE” framework for the design and delivery of organised PA sessions for children and adolescents [70]. Our findings support this principle in relation to MVPA participation during PE lessons. This is of significance due to the importance of PE within the school environment; research has shown that PE plays a considerable role in providing PA for children with increased activity levels on days in which PE is provided [18]. Furthermore, PE can develop fitness, gross motor skills and overall health [16]. PA provision scores obtained by schools also significantly predicted PE MVPA levels. In the context of UK schools there is a need for an objective measure, which captures how schools operate in relation to PA provision, as opposed to the US based tools previously published [54,55]. Within UK schools government funding is provided to improve the quality and breadth of PE and sports provision in primary schools worth £150 million per year [71]. Whilst not exclusively for PE delivery, UK schools have the freedom to determine how best to use this funding to improve curricular and non-curricular PA provision, but are expected to be accountable for measuring the impact of their spending [71]. Elsewhere, such as in the U.S., school based PA opportunities differ from state to state, district to district and from school to school based on decisions made by state policy makers [72]. Local policies and the degree to which they are adhered to or enforced there, impacts children’s PA accrual in schools [54]. Given the differences between school operations in these examples of the UK and U.S., objective tools to measure school based PA provision which are country-specific would be useful to help schools decide on how to use funding or to help policy makers understand what is being done at the level of individual schools. Furthermore, the use of an objective tool would be useful for researchers who wish to implement school-based interventions targeting areas of the school day most in need of intervention. In our analyses, school-level variables had limited associations with ST, LPA, or MVPA. Furthermore, PA provision scores from the audit tool did not explain or capture the differences between schools. Variance of activity levels explained by differences between schools were substantial, suggesting behaviours during periods of the school day varied between the participating schools. For example 54% of morning break and 75% of lunch break ST variance was explained by differences between schools. In comparison, a study examining children’s ST and MVPA during recess found total variance explained by differences between schools to be 12% for ST [73]. It is unclear why the between-school variance is higher than was reported by Ridgers et al. (2010) [73], particularly for ST. There are however a range of different factors related to school break times which can vary between individual schools. The current analyses included PA provision, playground space, and number of children, while other studies have shown provision of equipment, climate, and number of permanent play facilities to be associated with PA behaviour [73,74]. Thus, differences such as these which are particular to individual schools impact children’s ST and PA, and serve to highlight the need for analyses to account for the contribution of schools to PA outcome variance.

Number of children on roll inconsistently predicted ST and PA, depending on the period. For example, at morning break number on roll predicted more ST and LPA, whilst at lunch break it was associated with more ST and MVPA. A review of the overall PA behaviour of 10–18 year olds found the presence of peers and friends to be associated with PA [75]. This is to be expected in contexts such as morning break and lunch break, particularly in younger age groups, as peers will always be present. A systematic review of PA during school recess found 48 studies that reported a negative association between number on roll and PA and 38 studies reporting no association [76]. Given the inconsistencies of the current study and that of previous research, methodologies such as context-specific systematic observations and tools (e.g., SOCARP) [77] would help to further our understanding of children′s PA-related social dynamics and behaviours.

The subjective nature of the audit tool used and its completion by school staff is a limitation of the current study. A further limitation was the use of timetabled school times to define the segments of break and lunch times and PE. Actual recording of specific school period times during monitor wear by teachers would allow greater certainty that the activity recorded took place in the period of interest. This though would place additional burden on class teachers to record these times on multiple occasions each day. A greater range of school-level predictors may have better explained differences between schools, for example the presence of equipment during break and lunch breaks, fixed equipment and playground markings. The most important limitation is the cross-sectional nature of the research design which prevents conclusions to be made regarding causality. A strength of this study was the use of objectively assessed PA. Furthermore, the use of raw accelerations avoids the uncertainty of pre-processed data such as counts and the possibility that signal filtering methods alter study results [78,79]. The use of raw data also gives an increased control over data processing as well as the opportunity to improve comparability and consistency between studies which use different monitors for example [51]. In addition, the multilevel analyses allowed for the nested nature of children within schools and also school level correlates to be studied.

## 5. Conclusions

The most consistent child-level predictors of behaviour were levels of MVPA and ST, and maturity offset. School-level predictors were more inconsistent but included of children on roll and playground area. Understanding school-level variables which influence PA would be useful for both schools and researchers who wish to increase school based PA. The school environment is of great importance for PA promotion in children, which is exemplified by the UK government′s aim for children to accrue 30 min of MVPA during the school day [30]. Future research should consider setting-specific PA and its correlates/predictors within specific school days contexts.

## Figures and Tables

**Table 1:** Descriptive characteristics of participating children (Mean (SD) unless stated).

Characteristics	Boys (*n* = 110)	Girls (*n* = 105)
Age (year)	10.2 (0.3)	10.2 (0.3)
Stature (cm)	140.4 (5.9)	141.3 (6.8)
Body Mass (kg)	36.4 (8.4)	38.3 (10.6)
BMI (kg·m^2^)	18.9 (4.0)	18.3 (3.2)
BMI z-score	0.5 (1.3)	0.5 (1.3)
Weight Status		
Normal Weight (%)	76.2	72.5
Overweight/Obese (%)	23.8	27.5
Waist Circumference (cm)	64.3 (10.0)	64.9 (10.3)
Maturity Offset (y)	−2.8 (0.3)	−1.6 (0.4)
IMD Rank	5746.5 (5831.6)	6077.6 (6922.1)
IMD Decile	2.3 (1.7)	2.4 (2.1)
CRF (Number of shuttles)	30.4 (16.5)	25.4 (11.7)

CRF, cardiorespiratory fitness.

**Table 2:** Boys′ and girls′ sedentary time and physical activity (Mean and SD).

Time Segments	Boys (*n* = 92)			Girls (*n* = 94)		
	ST	LPA	MVPA	ST	LPA	MVPA
Weekday	546.7 (115.6)	357.5 (62.8)	42.0 (17.6) ^‡^	553.4 (108.8)	370.6 (55.6)	30.2 (13.4) ^‡^
School day	198.4 (31.3) ^†^	157.5 (27.4)	20.9 (8.7) ^‡^	210.4 (32.6) ^†^	151.9 (27.9)	14.3 (7.2) ^‡^
Morning break	6.4 (3.0)	7.2 (2.0)	1.5 (1.1) ^‡^	6.5 (3.1)	7.0 (2.0)	0.8 (0.06) ^‡^
Lunch break	17.0 (6.2) ^†^	7.0 (2.2) ^†^	6.0 (4.4) ^‡^	19.8 (8.0) ^†^	6.0 (2.0) ^†^	3.1 (2.2) ^‡^
PE	17.1 (8.1)	34.0 (5.7)	7.3 (4.1) ^†^	18.6 (8.5)	33.5 (6.6)	5.9 (3.6) ^†^

ST, sedentary time; LPA, light physical activity; MVPA, moderate to vigorous physical activity. ^†^ Significant difference between sexes, *p* < 0.05. ^‡^ Significant difference between sexes, *p* < 0.001.

**Table 3:** Descriptive school level predictors.

Variable	Mean (SD)	Range
No. enrolled students	277.6 (150.5)	102–579
Playground area (m^2^)	2071.6 (815.5)	904–3121
PA provision score (%)	62.3 (9.5)	52–75

**Table 4:** Multilevel associations between child and school level predictors and school day sedentary time and physical activity.

Correlate	School Day ST		School Day LPA		School Day MVPA	
	β (SE) ^1^	95% CI	β (SE)	95% CI	β (SE)	95% CI
Constant	**235.65 (5.92) ^‡^**	224.05 to 247.25	**354.0 (7.12) ^‡^**	340.04 to 368.0	**24.01 (5.0) ^‡^**	14.21 to 33.81
Child level variables						
Maturity Offset (y)	NE ^2^		NE		**−3.26 (0.60) ^‡^**	−4.44 to −2.08
CRF (total shuttles)	NE		**−0.07 (0.03) ^†^**	−0.13 to −0.01	**0.06 (0.03) ^‡^**	0.00 to 0.12
School day ST	NE		**−0.87 (0.02) ^‡^**	−0.91 to −0.83	**−0.11 (0.02) ^‡^**	−0.15 to −0.07
School day MVPA	**−1.92 (0.21) ^‡^**	−2.33 to −1.51	NE		NE	
Out of school MVPA	NE		**−0.32 (0.07) ^‡^**	−0.46 to −0.18	**0.25 (0.06) ^‡^**	0.13 to 0.37
School level variables						
No. on roll	NE		**−0.04 (0.02) ^†^**	−0.08 to −0.00	NE	
Playground area (m^2^)	NE		NE		**0.002 (0.00) ^†^**	0.00 to 0.00
School level variance	138.12 (84.44)		47.34 (26.22)		6.12 (3.91)	
Child level variance	419.16 (44.31)		30.60 (3.26)		25.29 (2.73)	
ICC	0.25		0.61		0.19	

^1^ Beta values reflect differences in minutes of ST/LPA/MVPA for every 1 measured unit of each predictor variable. ^2^ NE = not entered in final model. ST, sedentary time; LPA, light physical activity; MVPA, moderate to vigorous physical activity; CRF, cardiorespiratory fitness; ICC, intraclass correlation coefficient. ^†^ *p* < 0.05, ^‡^ *p* < 0.001.

**Table 5:** Multilevel associations between child and school level predictors and morning break sedentary time and physical activity.

Correlate	Morning Break ST		Morning Break LPA		Morning Break MVPA	
	β (SE) ^1^	95% CI	β (SE)	95% CI	β (SE)	95% CI
Constant	**3.83 (1.13) ^‡^**	1.62 to 6.04	**12.52 (1.07) ^‡^**	10.42 to 14.62	**2.33 (0.49) ^‡^**	1.37 to 3.29
Child level variables						
Maturity Offset (y)	NE ^2^		NE		**−0.36 (0.11) ^‡^**	−0.58 to −0.14
Weight Status ^3^	NE		NE		**−0.28 (0.12) ^†^**	−0.52 to −0.05
School day ST	NE		**−0.04 (0.00) ^‡^**	−0.04 to −0.03	**−0.01 (0.00) ^‡^**	−0.01 to −0.00
School day MVPA	**−0.07 (0.01) ^‡^**	−0.09 to −0.05	NE		NE	
Out of school MVPA	NE		**−0.03 (0.01) ^‡^**	−0.05 to −0.01	NE	
School level variables						
No. on roll	**0.01 (0.00) ^††^**	0.00 to 0.02	**0.007 (0.00) ^‡^**	0.00 to 0.01	NE	
School level variance	1.77 (0.98)		0.65 (0.39)		0.0 (0.04)	
Child level variance	1.52 (0.16)		1.42 (0.15)		0.43 (0.05)	
ICC	0.54		0.31		0.00	

^1^ Beta values reflect differences in minutes of ST/LPA/MVPA for every 1 measured unit of each predictor variable. ^2^ NE = not entered in final model. ^3^ Reference group for weight status was normal weight. ST, sedentary time; LPA, light physical activity; MVPA, moderate to vigorous physical activity; ICC, intraclass correlation coefficient. ^†^ *p* < 0.05, ^††^ *p* < 0.01, ^‡^ *p* < 0.001.

**Table 6:** Multilevel associations between child and school level predictors and lunch break sedentary time and physical activity.

Correlate	Lunch Break ST		Lunch Break LPA		Lunch Break MVPA	
	β (SE) ^1^	95% CI	β (SE)	95% CI	β (SE)	95% CI
Constant	**10.70 (4.95) ^††^**	1.0 to 20.4	**17.77 (1.12) ^‡^**	15.57 to 19.97	**8.98 (2.43) ^‡^**	4.13 to 13.74
Child level variables						
WtHR	NE ^2^		NE		**−9.28 (2.96) ^‡^**	−15.08 to −3.48
School day ST	NE		**−0.06 (0.01) ^‡^**	−0.08 to −0.04	**−0.03 (0.00) ^‡^**	−0.05 to −0.02
School day MVPA	**−0.33 (0.04) ^‡^**	−0.09 to −0.05	NE		NE	
Out of school MVPA	NE		NE		**0.09 (0.03) ^††^**	0.03 to 0.15
School level variables						
No. on roll	**0.04 (0.02) ^†^**	0.00 to 0.02	NE		**0.02 (0.00) ^‡^**	0.001 to 0.03
School level variance	33.45 (18.16)		2.72 (1.52)		1.50 (0.96)	
Child level variance	11.4 (1.2)		2.25 (0.24)		6.24 (0.66)	
ICC	0.75		0.55		0.19	

^1^ Beta values reflect differences in minutes of ST/LPA/MVPA for every 1 measured unit of each predictor variable. ^2^ NE = not entered in final model. ST, sedentary time; LPA, light physical activity; MVPA, moderate to vigorous physical activity; WtHR waist to height ratio; ICC, intraclass correlation coefficient. **^†^** *p* < 0.05, **^††^** *p* < 0.01, **^‡^** *p* < 0.001.

**Table 7:** Multilevel associations between child and school level predictors and PE sedentary time and physical activity.

Correlate	PE ST		PE LPA		PE MVPA	
	β (SE) ^1^	95% CI	β (SE)	95% CI	β (SE)	95% CI
Constant	**21.58 (2.48) ^‡^**	16.72 to 26.44	**54.84 (3.74) ^‡^**	47.51 to 62.17	2.80 (2.63)	−2.35 to 7.95
Child level variables						
Maturity Offset (y)	NE ^2^		NE		**−0.99 (0.29) ^‡^**	−1.56 to −0.42
Weight Status ^3^	NE		**2.15 (0.83) ^††^**	0.52 to 3.78	NE	
PA Enjoyment	NE		NE		**1.22 (0.34) ^‡^**	0.55 to 1.89
School day ST	NE		**−0.10 (0.01) ^‡^**	−0.12 to −0.08	**−0.02 (0.00) ^††^**	−0.04 to −0.01
School day MVPA	**−0.29 (0.06) ^‡^**	−0.41 to −0.17	NE		NE	
Out of school MVPA	NE		**−0.12 (0.05) ^†^**	−0.22 to −0.02	0.13 (0.03) ^‡^	0.07 to 0.19
School level variables						
School level variance	33.55 (18.90)		26.91 (14.87)		6.55 (3.66)	
Child level variance	35.67 (3.78)		20.31 (2.15)		5.86 (0.63)	
ICC	0.48		0.57		0.53	

^1^ Beta values reflect differences in minutes of ST/LPA/MVPA for every 1 measured unit of each predictor variable. ^2^ NE = not entered in final model. ^3^ Reference group for weight status was normal weight. ST, sedentary time; LPA, light physical activity; MVPA, moderate to vigorous physical activity; ICC, intraclass correlation coefficient. ^†^ *p* < 0.05, ^††^ *p* < 0.01, ^‡^ *p* < 0.001.
